# A single-arm pilot study of metformin in patients with autosomal dominant polycystic kidney disease

**DOI:** 10.1186/s12882-019-1463-2

**Published:** 2019-07-23

**Authors:** Bogdan Marian Sorohan, Gener Ismail, Andreea Andronesi, Georgia Micu, Bogdan Obrișcă, Roxana Jurubiță, Ioanel Sinescu, Cătălin Baston

**Affiliations:** 1 Carol Davila University of Medicine and Pharmacy, Bucharest, Romania; 2 Nephrology Department, Fundeni Clinical Institute, Fundeni Street No. 258, ZIP Code 022328, District No.2, Bucharest, Romania; 3 Center of Uronephrology and Renal Transplantation, Fundeni Clinical Institute, Bucharest, Romania

**Keywords:** Autosomal dominant polycystic kidney disease, Metformin, Glomerular filtration rate, Body mass index, Tolerability

## Abstract

**Background:**

Metformin has shown promising results regarding cystogenesis inhibition in preclinical studies with autosomal dominant polycystic kidney disease (ADPKD) models. We designed a prospective, preliminary, single-arm study to evaluate the tolerability, safety and the effect of Metformin on kidney function and body mass index (BMI) in Romanian patients with ADPKD.

**Methods:**

We enrolled 34 adult patients with ADPKD, chronic kidney disease (CKD) stages 1–5 not on dialysis and without diabetes mellitus. The primary endpoint was to assess the tolerability and safety of Metformin. The secondary endpoints evaluated changes in estimated glomerular filtration rate (eGFR), body mass index (BMI) and renal replacement therapy (RRT) necessity. Patients received an initial dose of Metformin of 500 mg/day within the first month that was increased to 1000 mg/day thereafter according to tolerability. Change in eGFR and BMI was expressed as mean difference with the corresponding 95% confidence intervals and as a percentage. For the primary endpoint, we included all 34 enrolled patients. To assess the secondary endpoint, intention-to-treat (ITT) and per-protocol (PP) analysis was performed.

**Results:**

Sixteen patients out of 34 completed the follow-up period at 24 months. Eighteen patients developed adverse events and 63.6% of these events were gastrointestinal related. Nausea was the most common adverse event (17.6%). Two patients (5.8%) permanently discontinued medication due to adverse events. We recorded no case of hypoglycemia, lactic acidosis or death. Mean eGFR changed by − 1.57 ml/min/1.73m^2^ (95%CI:-22.28 to 19.14, *P* = 0.87) in ITT and by − 4.57 ml/min/1.73m^2^ (95%CI:-28.03 to 18.89, *P* = 0.69) in PP population. Mean BMI change was − 1.10 kg/m^2^ (95%CI:-3.22 to 1.02, *P* = 0.30) in ITT population and − 0.80 kg/m^2^ (95%CI:-3.27 to 1.67, *P* = 0.51) in PP analysis. Three patients (8.8%) needed RRT.

**Conclusions:**

Metformin was well tolerated, had a good safety profile even in ADPKD patients with advanced CKD and it was not associated with change in eGFR or BMI across the follow-up period.

**Trial registration:**

The study was retrospectively registered on https://www.isrctn.com (number ISRCTN 93749377); date registered: 02/25/2019.

## Background

Autosomal Dominant Polycystic Kidney Disease (ADPKD) is the most common hereditary kidney disease, characterized by multiple, bilateral renal cysts leading to an increased kidney volume and progressive loss of kidney function. It is a systemic disorder that also affects the liver, pancreas, spleen, seminal vesicles, arachnoid membrane and endothelium [1]. Recent epidemiologic studies reported an average prevalence of ADPKD in Europe of 2.7:10,000 (95% CI = 0.73–4.67) and a point prevalence of 3.96:10,000 (95% CI = 0.94–0.98) in countries from the European Union [2, 3]. According to the European Renal Association-European Dialysis and Transplant Association (ERA-EDTA) Registry, the estimated prevalence of ADPKD in Romania was 1.9:10,000 in 2012 [3]. PKD 1 and PKD 2 genes mutations are involved in the majority of cases, but recently new gene players were described, such as GANAB and DNAJB11 [4, 5]. Polycystin 1 and 2 encoded by PKD genes are expressed mainly in tubular epithelial cells and work physiologically as an unit with well defined functional and structural roles [6]. In ADPKD, the abnormality of polycystin 1 and 2 proteins leads to primary cilia dysfunction, activation of several intracellular pathways, increased fluid secretion, cell-cell adhesion issues and hyperproliferation. Among the most important intracellular pathways involved were cyclic adenosine monophosphate (cAMP), mechanistic target of rapamycin (mTOR), mitogen-activated protein kinase/extracellular signal-regulated kinase (MAPK/ERK), adenosine monophosphate-activated protein kinase (AMPK), just another kinase/signal transducer and activator of transcription (JAK/STAT) [7]. During the last decade, different therapeutic agents were used to counteract cyst and kidney dysfunction progression [8]. Metformin, the most common used biguanide for the treatment of type 2 diabetes mellitus, with a good safety profile, has shown promising results regarding cystogenesis inhibition in preclinical studies [9, 10]. The proposed mechanism of action in ADPKD is by stimulating AMPK activation, which negatively regulates both the chloride channel cystic fibrosis transmembrane conductance regulator (CFTR) from the apical membrane and mTOR pathway. CFTR activity is directly inhibited and that of the mTOR pathway is indirectly inhibited through phosphorylation protein of tuberous sclerosis 2 (TSC2) and Raptor, leading to the blockage of two essential processes of cystogenesis, fluid secretion and proliferation [9]. Evolution of kidney function in patients with ADPKD is characterized by a high individual variability. Several environmental, genetic, demographic, clinical, biological and structural factors were associated with disease progression [11]. Among them, overweight and particularly, obesity were associated with greater kidney function decline and disease progression [12]. It has already been demonstrated that Metformin promotes weight loss in overweight and obese patients [13]. Apart from the direct effect on cystogenesis, Metformin could have a beneficial additional effect in controlling the decline of renal function by producing a decrease in body mass index (BMI).

On the basis of these observations, we designed a prospective, preliminary, single-arm study to evaluate the tolerability, safety and the effect of Metformin on kidney function and BMI in Romanian patients with ADPKD.

## Methods

### Study design

METformin in ROmanian Patients with autosomal dominant polycystic kidney disease (METROP) was designed as a pilot, single center, interventional, single-arm study to investigate the safety, tolerability of Metformin and the change in kidney function and BMI under treatment, in Romanian adult patients with ADPKD.

### Inclusion and exclusion criteria

**Fig. 1:**
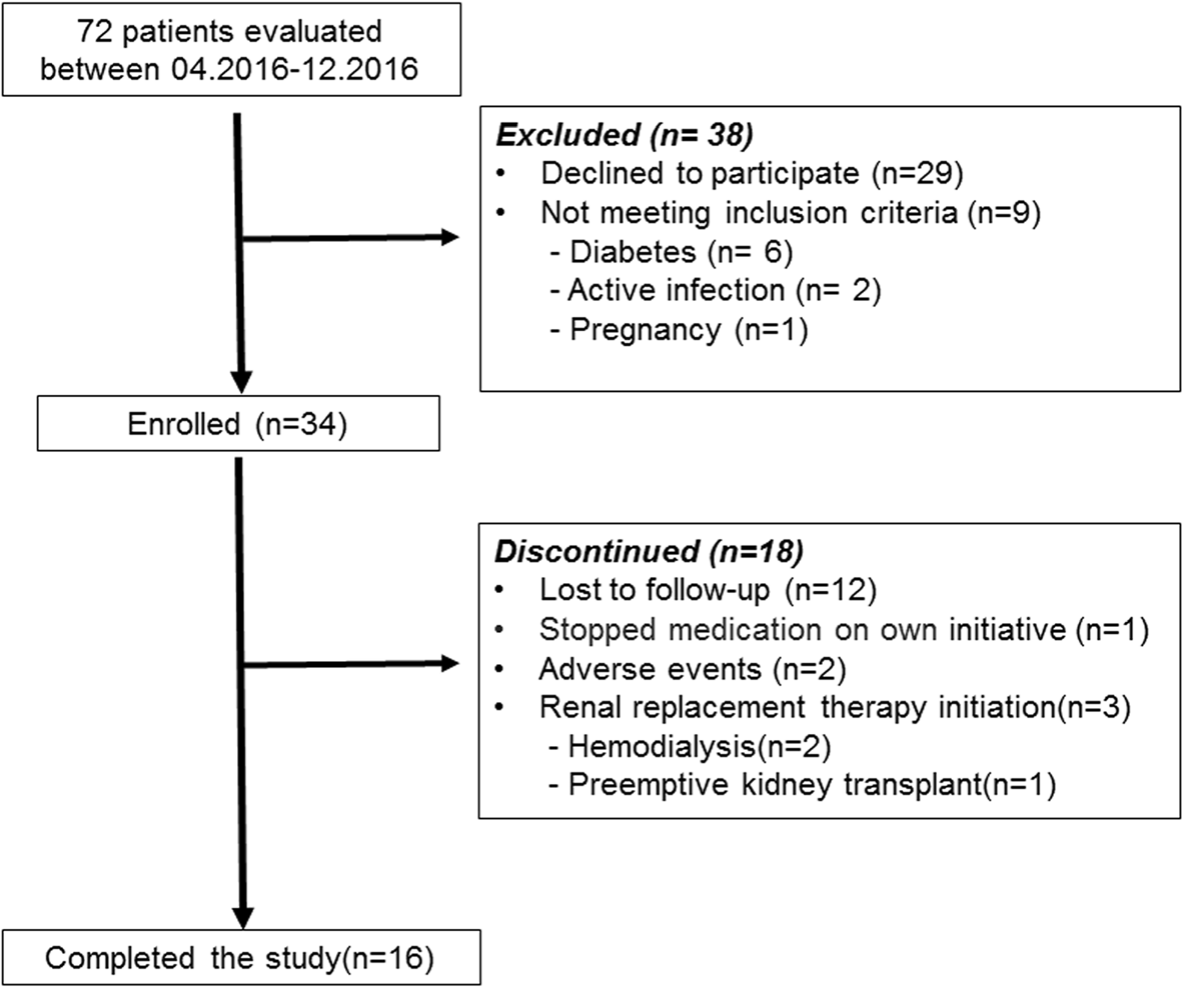
Study flow diagram

Between April 2016 and December 2016, 34 patients were enrolled (Fig. 1). Inclusion criteria were: adult patients (age ≥ 18 years), diagnosis of ADPKD based on unified ultrasonographic Pei-Ravine criteria [14], CKD stages between 1 and 5 not on dialysis. We ruled out: patients with diabetes mellitus, any patient with active infections, pregnant or breastfeeding patients, those with known contraindication or allergy to Metformin and those on renal replacement therapy (RRT)*.*

### Endpoints

The primary endpoint was to assess the tolerability and safety of Metformin in patients with ADPKD. The tolerability outcome included evaluation of the number and type of gastrointestinal and non-gastrointestinal symptoms. The safety outcome analyzed the presence of hypoglycemia, lactic acidosis, death and other adverse events. Also, the percentage of patients that permanently discontinued medication due to adverse events was studied.

The secondary endpoints evaluated the change in kidney function from baseline, number/percentage of patients that needed RRT and change in BMI from the baseline value after 12 and 24 months of treatment with Metformin.

### Metformin dosage

Patients received an initial dose of Metformin of 500 mg/day within the first month, that was increased to 1000 mg/day (500 mg twice daily), depending on tolerance and adverse events. Drug initiation and dose augmentation were made in the hospital. If patients declared that medication was well tolerated, with no severe gastrointestinal symptoms and if no hypoglycemia or lactic acidosis was found in the laboratory tests, the dose was increased to 1000 mg/day after the first month. The dose of Metformin in patients with CKD stage 5 was limited to 500 mg/day throughout the duration of the study.

### Study follow-up and data collection

**Fig. 2:**
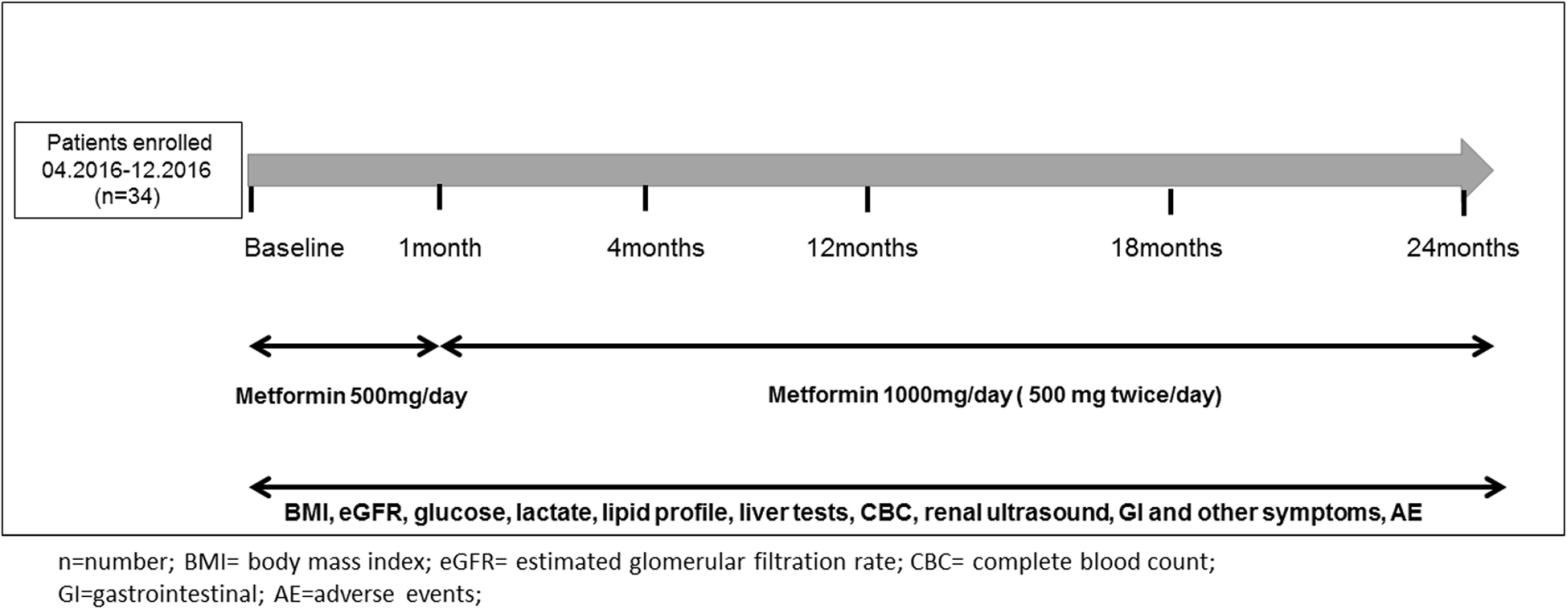
Study enrollment, follow-up, patient evaluation and medication dose

The study follow-up period was of 24 months. In the first year, visits were established at 1, 4 and 12 months, and after this period, at 18 and 24 months. At baseline, data regarding personal medical history, family history of ADPKD, demographic, smoking status and antihypertensive drugs were collected. Also, at baseline and at each study visit, patients were questioned about drug tolerability, underwent physical examination, including BMI assessment and laboratory tests were performed, including: glycemic and lipid profiles, liver tests, renal function tests, lactic acid levels, complete blood count and urinary tests (Fig. 2).

### Safety, tolerability, kidney function and body mass index

Gastrointestinal tolerability of Metformin was assessed by evaluating the occurrence rates of symptoms such as nausea, vomiting, diarrhea, bloating, abdominal pain and non-gastrointestinal related symptoms. To assess the severity of the GI symptoms, we used the Gastrointestinal Symptoms Rating Scale which consisted of 15 items, each rated on a 7-point Likert scale from no discomfort to very severe discomfort, conducted in an interview manner by a single physician [15]. For the safety analysis, hypoglycemia was defined as blood glucose < 70 mg/dl with or without typical adrenergic symptoms and lactic acidosis was defined as lactic acid level > 4 mmol/L obtained from venous samples. Mortality was defined as the number of patients who died during the study follow-up. Other adverse events were reported as any adverse event that occurred during the treatment with Metformin. Metformin was permanently discontinued in case of severe gastrointestinal and non-gastrointestinal symptoms, symptomatic hypoglycemia and lactic acidosis. In case of mild/moderate persistent gastrointestinal and non-gastrointestinal symptoms, the dose was decreased until tolerance was obtained. Renal function was evaluated based on serum creatinine, estimated by CKD-EPI formula and expressed as estimated glomerular filtration rate (eGFR). BMI was calculated based on kilograms divided by the square of height in meters (kg/m^2^). A BMI between 25 and 29.9 kg/m^2^ falls within the overweight range and > 30 kg/m^2^ in the obese range.

### Statistical analysis

Variables were summarized using descriptive statistics as percentages for binary variables, mean ± standard deviation for continuous normally distributed and median with interquartile range for continuous abnormally distributed variables. Change in eGFR and BMI was expressed as mean difference with the corresponding 95% confidence intervals and as a percentage. For the primary endpoint, we included all 34 enrolled patients. To assess the secondary endpoint, intention-to-treat (ITT) and per-protocol (PP) analysis was performed. ITT population included all enrolled patients (34 patients), regardless of treatment discontinuation or protocol violation. PP population (16 patients) included only those patients who adhered to protocol. *P* value < 0.05 was considered statistically significant. Statistical analysis was performed using IBM SPSS 20 Software (Chicago, Illinois).

## Results

### Patients

A total of 34 patients with ADPKD were enrolled in the study. Sixteen (47%) out of 34 patients completed the follow-up period at 24 months. Of those who did not adhere to the protocol, 12 (66.7%) were lost to follow-up, 1 patient (5.6%) stopped the medication by his own, in 2 patients (11.1%) medication was withdrawn because of adverse events and 3 patients (16.6%) needed renal replacement therapy (Fig. 1).

### Baseline characteristics

Among the 34 patients, mean age at baseline was 47.29 ± 11.29 years, the majority of patients were females (64.7%) and the mean age at diagnosis was 35.97 ± 11.66 years. Only 6 patients out of 34 did not have a family history of ADPKD. Mean BMI was 27.10 ± 3.65 kg/m^2^, 50% of patients were overweight and 23.5% were obese, 76.5% were hypertensive, 67.6% were dyslipidaemic and 29.4% were current smokers. Mean eGFR at baseline was 63.75 ± 34.71 ml/min/1.73m^2^ and 67.6% of patients had CKD stages 1–3. Urinalysis showed that patients were characterized by hypostenuria. Twenty-one subjects (61.8%) were under blood-pressure control medication, 18 out of them receiving angiotensin converting enzyme inhibitors or angiotensin II receptor blockers (Table 1).

**Table 1:** Baseline characteristics of study population

Variables	Patients number (*N* = 34)
Age (mean, years)	47.29 ± 11.29
Gender (%)	
Male	12 (35.3%)
Female	22 (64.7%)
Family history of ADPKD (%)	28 (82.4%)
Mother related	11 (32.4%)
Father related	17 (50%)
None	6 (17.6%)
Age at diagnosis (mean, years)	35.97 ± 11.66
BMI (mean, kg/m^2^)	27.10 ± 3.65
25–29.9 (%)	17 (50%)
≥ 30 (%)	8 (23.5%)
Smoking status (%)	
Current smoker	10 (29.4%)
Past smoker	6 (17.6%)
Non-smoker	18 (52.9%)
History of hematuria (%)	4 (11.8%)
History of kidney stones (%)	12 (35.3%)
History of lumbar pain (%)	28 (82.4%)
History of UTI (%)	14 (44.1%)
HTN (%)	26 (76.5%)
Dyslipidemia (%)	23 (67.6%)
SBP (mean, mmHg)	136.18 ± 15.42
DBP (mean, mmHg)	83.09 ± 11.54
Serum creatinine (median, mg/dl)	1.11 (0.88–2.61)
eGFR (mean, ml/min/1.73m^2^)	60.45 ± 34.71
CKD stage (%)	
1	8 (23.5%)
2	10 (29.4%)
3	5 (14.7%)
4	6 (17.6%)
5	5 (14.7%)
Serum urea (median, mg/dl)	59 (36.42–80.75)
Glucose level (mean, mg/dl)	87.75 ± 15.90
Total cholesterol (mean, mg/dl)	186.88 ± 37.32
Triglycerides (median, mg/dl)	95.50 (70–145.25)
Antihypertensive treatment (%)	21 (61.8%)
ACEI/ARB (%)	18 (52.9%)
Urine specific gravity (mean)	1012 ± 6.39

*N* number, *ADPKD* autosomal dominant polycystic kidney disease, *BMI* body mass index, *UTI* urinary tract infections, *HTN* arterial hypertension, *SBP* systolic blood pressure, *DBP* diastolic blood pressure, *GFR* glomerular filtration rate, *CKD* chronic kidney disease, *ACEI* angiotensin converting enzyme inhibitors, *ARB* angiotensin II receptor blockers

### Safety analysis

During the 24 months of follow-up, 18 patients developed adverse events, and 63.6% of the events were gastrointestinal related. The most common gastrointestinal event was nausea, which appeared in 6 patients (17.6%). One patient developed both vomiting and diarrhea. Of the non-gastrointestinal related adverse events, we observed renal cyst complications in 4 patients (11.7%), including one patient with hemorrhage associated with cysts infection that required antibiotic therapy and one with intracystic hemorrhage. Among other non-gastrointestinal related events, we could pointed out rash in one patient and dizziness in another patient. Six of the 18 patients that experienced adverse events (33.3%) developed them in the first month of treatment, at the initial dose of 500 mg per day. Adverse events that occurred after the first month of treatment, when the dose was increased at 1000 mg per day were: nausea (4 patients), 2 cases of abdominal pain (2 patients), bloating (2 patients) and renal cyst complications (4 patients). Nausea and dizziness, which appeared within the first month of follow-up, were mild and temporary and did not limit the increase of Metformin dose. During follow-up, no adverse event has determined the decrease of dose, except for the cases where permanently discontinuation was necessary. In our study population, we did not observe any case of hypoglycemia or lactic acidosis and no patient died during the follow-up period (Table 2).

**Table 2:** Adverse events during the study period

Adverse event type	No. of patients with event (%)
Gastrointestinal related	
Nausea	6 (17.6%)
Vomiting	1 (2.9%)
Diarrhea	1 (2.9%)
Abdominal pain	2 (5.8%)
Bloating	2 (5.8%)
Non-gastrointestinal related	
Dizziness	1(2.9%)
Rash	1 (2.9%)
Renal cyst complications (infection and/or hemorrhage)	4 (11.7%)
Lactic acidosis	0 (0%)
Hypoglycemia	0 (0%)
Death	0 (0%)

Metformin was permanently discontinued in 2 patients (5.8%) due to adverse events. In one patient, the reason for discontinuation was severe abdominal pain, which appeared after 4 months of follow-up, at the dosage of 1000 mg/day. Regarding the other patient, we stopped Metformin because of severe gastrointestinal and skin manifestations, including vomiting, diarrhea and rash, which appeared during the second week, at the initial dose of 500 mg per day.

### Secondary endpoints analysis

#### Change in estimated glomerular filtration rate over 24 months of treatment

Evolution of renal function in ITT population is shown in Table 3a. The observed change of mean eGFR after 4, 12 and 24 months of treatment was of + 1.71 ml/min/1.73m^2^ (95% CI: − 16.56 to 19.98; *P* = 0.85), − 1.03 ml/min/1.73m^2^ (95% CI: − 21.28 to 19.22, *P* = 0.91) and − 1.57 ml/min/1.73m^2^ (95% CI: − 22.28 to 19.14, *P* = 0.87), respectively.

**Table 3:** Evolution of eGFR and BMI over 24 months of treatment in intention-to-treat and per-protocol population

No. of patients	Study period	Mean eGFR value (ml/min/1.73m^2^)	Mean eGFR change (ml/min/1.73m^2^)	95% CI	*P Value*	Mean BMI Value (kg/m^2^)	Mean BMI change (kg/m^2^)	95% CI	*P Value*
A. Mean eGFR and BMI changes in intention to treat population									
34	Baseline	60.45 ± 34.71	–	–	–	27.10 ± 3.65	–	–	–
28	1 month	61.41 ± 34.17	0.96	−16.63 to −18.55	0.91	26.94 ± 3.58	−0.16	−2.00 to 1.68	0.86
25	4 months	62.16 ± 34.53	1.71	−16.56 to 19.98	0.85	26.45 ± 3.48	− 0.65	− 2.53 to 1.23	0.49
20	12 months	59.42 ± 37.67	−1.03	− 21.28 to 19.22	0.91	26.27 ± 3.32	−0.80	−2.82 to 1.16	0.40
16	24 months	58.88 ± 32.32	−1.57	−22.28 to 19.14	0.87	26.00 ± 3.07	−1.10	−3.22 to 1.02	0.30
B. Mean eGFR and BMI changes in per protocol population									
16	Baseline	63.45 ± 32.68	–	–	–	26.80 ± 3.76	–	–	–
16	1 month	64.83 ± 31.68	1.38	−21.85 to 24.61	0.90	26.56 ± 3.82	−0.24	−2.97 to 2.49	0.85
16	4 months	65.26 ± 30.65	1.81	−21.60 to 24.68	0.87	26.34 ± 3.60	−0.46	−3.11 to 2.19	0.76
16	12 months	62.80 ± 34.58	−0.65	−24.94 to 23.64	0.95	26.31 ± 3.46	−0.49	− 3.09 to 2.11	0.70
16	24 months	58.88 ± 32.32	− 4.57	−28.03 to 18.89	0.69	26.00 ± 3.07	−0.80	−3.27 to 1.67	0.51

*No*. number, *CI* confidence interval, *eGFR* estimated glomerular filtration rate, *BMI* body mass index

Evolution of renal function in per-protocol population is shown in Table 3b. The change from baseline of mean eGFR was of + 1.81 ml/min/1.73m^2^ (95% CI: − 21.60 to 24.68, P = 0.87) after the first 4 months, followed by − 0.65 ml/min/1.73m^2^ (95% CI: − 24.94 to 23.64, *P* = 0.95) at 12 months and − 4.57 ml/min/1.73m^2^ (95% CI: − 28.03 to 18.89, *P* = 0.69) at 24 months of treatment.

**Fig. 3:**
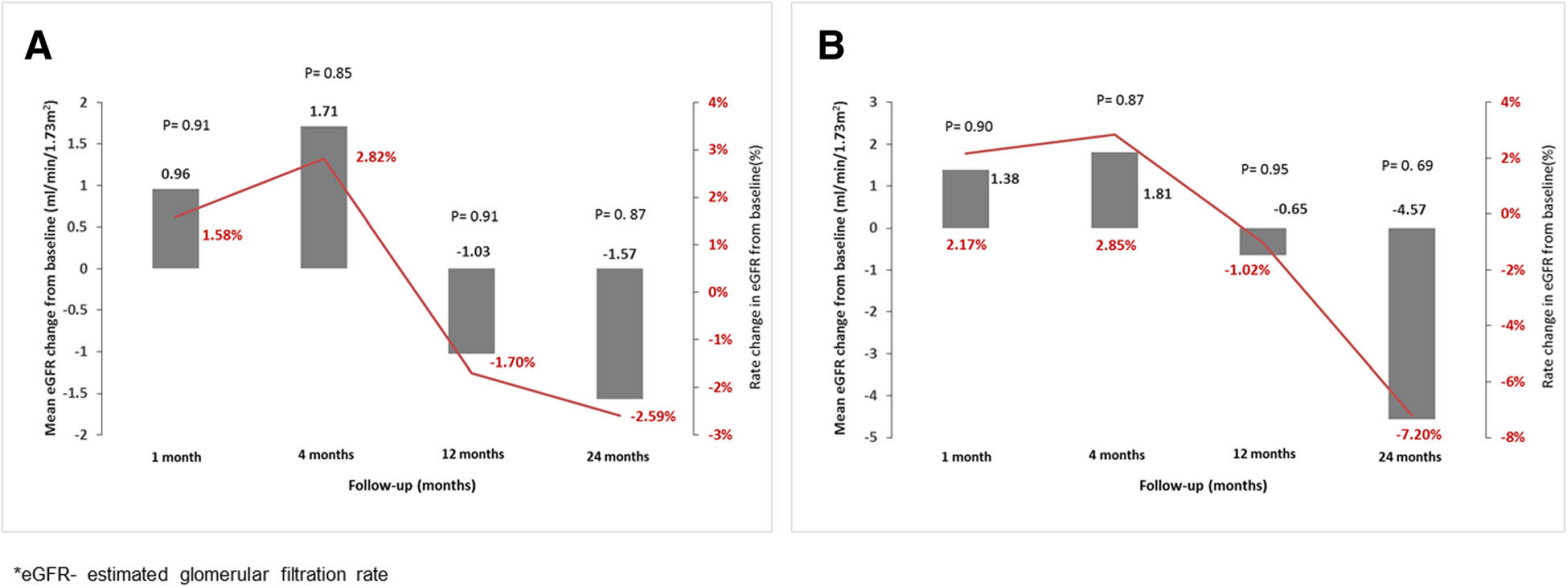
Evolution of eGFR over 24 months of treatment in intention-to-treat (**a**) and per-protocol analysis (**b**)

Rate changes of eGFR from baseline, corresponding to those of mean eGFR, were illustrated in Fig. 3a and b. After 24 months of treatment with Metformin, according to ITT analysis, eGFR had changed by − 2.59% and in agreement with PP analysis, by − 7.2%.

#### Change in body mass index over 24 months of treatment

**Fig. 4:**
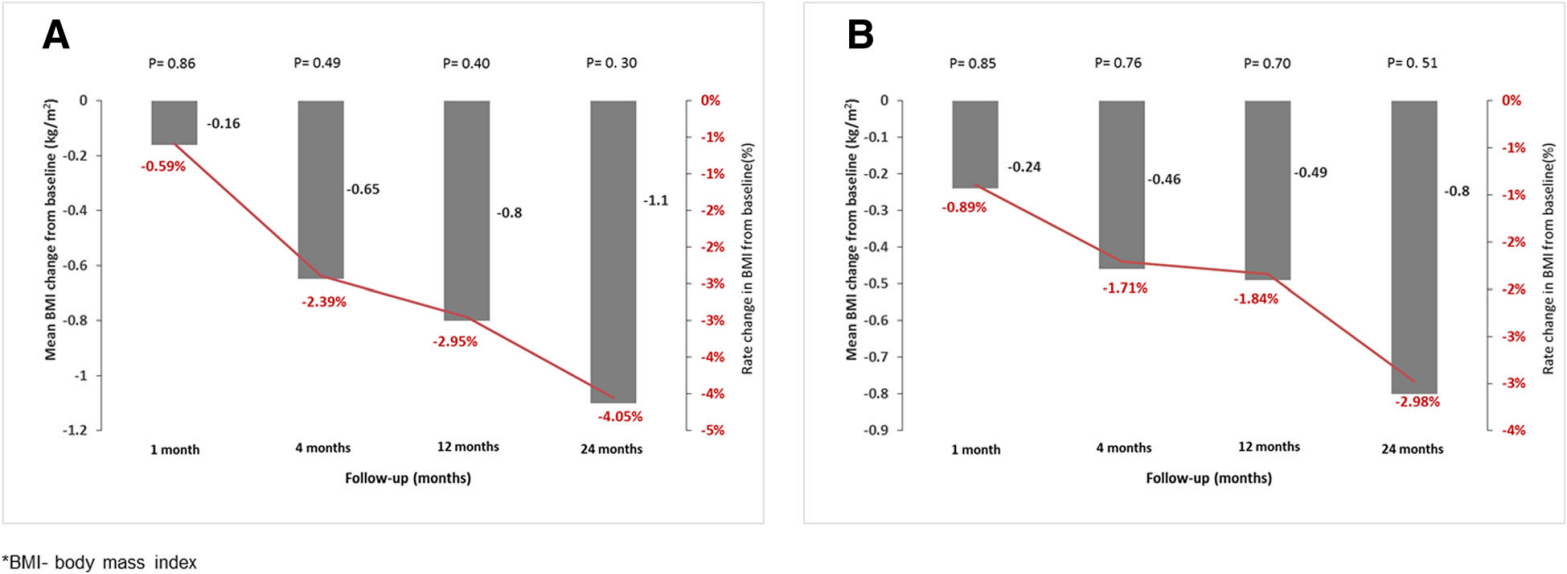
Evolution of BMI over 24 months of treatment in intention-to-treat (**a**) and per-protocol analysis (**b**)

Evolution of BMI is shown in Table 3a and b. At 24 months, mean BMI change from baseline was − 1.10 kg/m^2^ (95% CI:-3.22 to 1.02, *P* = 0.30) in ITT population and − 0.80 kg/m^2^ (95% CI: − 3.27 to 1.67, *P* = 0.51) in patients included in PP analysis. The rate changes of BMI from baseline value, concurrent with those of mean BMI, were of − 4.05% and − 2.98%, respectively (Fig. 4a and b).

#### Renal replacement therapy end-point

During the study period, 3 patients (8.8%) out of 34 needed renal replacement therapy. Of those, 2 became hemodialysis dependent and 1 patient underwent a preemptive living donor kidney transplantation. All patients were female, two had CKD stage 5 at the beginning of the study and received a dose of 500 mg/day of Metformin and the patient who underwent transplantation had CKD stage 4. None of these patients had a precipitating factor or an acute event before the RRT was started. The time from Metformin starting to RRT initiation was of 4 and 12 months for the two patients on hemodialysis and 12 months for the patient that underwent kidney transplantation.

## Discussion

Results from our study showed that treatment with Metformin was well tolerated in all stages of CKD, with few adverse events, and it was not associated with any changes in either eGFR or BMI at any time point. To our knowledge, this is the first report from a prospective, interventional study about Metformin in patients with ADPKD.

The most common adverse event found in our study was nausea (17.6%), but this manifestation was mild and transitory, no dose reduction or permanently discontinuation was required. This finding is in agreement with results from other studies, where nausea was among the most common adverse events in diabetic and non-diabetic patients treated with Metformin [16, 17]. It has been shown that the frequency of nausea was of 10% and that treatment discontinuation rate due to nausea was 3% in patients with type 2 diabetes treated with 1 g/day of Metformin [16]. Findings from our study do not exceed the current evidences regarding cyst infection in ADPKD patients and are in line with those of *Sallée M.* et al., who showed that 8.6% of patients developed cyst infection [18]. Hypoglycemia is a rare event in patients treated with Metformin, but the risk of hypoglycemia increases in those with CKD [19, 20]. In our study population, hypoglycemia was absent. The use of Metformin in patients with CKD is limited by guideline contraindications because of the fear for lactic acidosis. The European Medicines Agency and Food and Drug Association recommended to use Metformin up to an eGFR of 30 ml/min/1.73 m^2^, but this cut-off value was not based on results from prospective studies of pharmacodynamics and pharmacokinetics [21, 22]. The incidence of Metformin-associated lactic acidosis was 4.3 per 100,000 patient-years in a 2010 Cochrane analysis [23]. In this systematic review, *Salpeter* et al. demonstrated that Metformin was not associated with an increased risk of lactic acidosis compared to other antidiabetic drugs. Moreover, 53% of the studies included patients with CKD, including 37,360 patient-years with no events of lactic acidosis [23]. This data is supported by an observational cohort study from the Swedish National Diabetes Register, which analyzed 51,675 patients with type 2 diabetes mellitus, including 12.3% with eGFR ≤60 ml/min/1.73m^2^ and has shown that lactic acidosis is a rare event even in those with eGFR between 30 and 45 ml/min/1.73m^2^ [24]. *Duong* et al. reported no adverse events or a relationship between Metformin, lactic acid levels or lactic acidosis in 7 patients with advanced CKD, including 2 patients on dialysis, who were treated with lower doses of Metformin, between 250 and 500 mg/day [25]. Of note, a recent study by *Lalau* et al. showed that adjusting the dose of Metformin in patients with moderate-severe CKD seems to be safe and remained pharmacologically efficient. A dose of 500 mg/day does not determine a drug concentration > 5 mg/L, considered the safe upper limit, not even in CKD stages 4–5 and a dose of 1000 mg/day produced a drug concentration > 5 mg/L in 1 patient from CKD stages 4–5. Lactate concentration did not exceed 5 mmol/L in any patient [26]. In accordance with these findings, our study included patients in all stages of CKD and no case of lactic acidosis was reported.

Analysis of the kidney function showed no change in kidney function after 24 months of treatment, but with an initial increase within the first 4 months. This initial, transitory improvement in eGFR could be associated with the weight loss and subsequent change in BMI. It is well known that Metformin produces weight loss and BMI reduction even in overweight or obese patients without diabetes [13]. Moreover, one study has shown that weight loss leads to kidney function improvement, in those with baseline eGFR< 60 ml/min/1.73m^2^, especially in the first 6 months after dietary restriction [27]. In our study, the greater rate in BMI change occurred in the first 4 months (− 2.39%) and we also observed a concomitant variation in median serum creatinine (from 1.11 to 1.03 mg/dl) that could be influenced by muscle mass loss, thus affecting the eGFR value in this early period (mean eGFR change from baseline: + 1.71 ml/min/1.73m^2^). Two randomized control trials (NCT02656017; NCT02903511) involving Metformin safety and efficacy in ADPKD are ongoing. One retrospective, case-control study, which selected 7 diabetic ADPKD patients treated with Metformin and 7 matched non-diabetic ADPKD controls, evaluated the effect of Metformin on renal function progression during a 3 year follow-up. An overall crude loss of eGFR of – 0.9 ml/min/1.73m^2^ estimated by a linear mixed model at 36 months was reported in the Metformin group [28]. Our ITT analysis was in line with these results, as we found a change in mean eGFR of – 1.57 ml/min/1.73 m^2^ after 24 months of treatment. If we refer to other clinical trials testing medication to slow eGFR decline in ADPKD, in the TEMPO 3:4 study, which included patients with eGFR > 60 ml/min/1.73m^2^, Tolvaptan slowed the decline in kidney function after 36 months (− 2.61 vs − 3.81 [mg/ml]^− 1^ per year), but was associated with an increased rate of withdrawal due to adverse events [29]. REPRISE trial, which included patients with CKD stages 2–4, showed a mean eGFR change from baseline of − 2.34 ml/min/1.73 m^2^ in the Tolvaptan group, as compared with − 3.61 ml/min/1.73 m^2^ in the placebo group after 12 months (difference, 1.27 ml/minute/1.73 m^2^; 95% CI, 0.86 to 1.68; *P* < 0.001) [30]. The eGFR decline in these studies was higher than what we found at 12 and 24 months in ITT as well as in PP analysis. The efficacy of Sirolimus, an mTOR inhibitor, that partially shares some mechanisms of action with Metformin in cystogenesis inhibition, was tested in clinical trials. Despite the fact that SUISSE trial and the study of Braun et al. showed a favorable GFR evolution from baseline of + 0.2 ml/min/1.73m^2^ after 12 months (*P* = 0.07) and + 1.6 ml/min/1.73m^2^ after 18 months (P < 0.001) in the Sirolimus group, a meta-analysis demonstrated that mTORC1 inhibitors had no significant effect on eGFR change in patients with ADPKD (*P* = 0.22) [31–33].

In our study population, 73.5% were obese or overweight at baseline and we found a progressive decline in BMI, more accelerated within the first 4 months. *Nowak* et al. proved that obesity and overweight patients with ADPKD have a greater decline in eGFR that normal weight ones [12]. Obesity and ADPKD seem to share some common pathways. Positive energy balance, overnutrition and obesity can produce a hyperactivity of mTOR pathway via PI3k/Akt activation, may activate mTORC1/S6K and reduce AMPK activity. Hyperactivation of mTORC1/S6K pathway and decreased activity of AMPK are important players in ADPKD cystogenesis, regarding proliferation and cyst fluid secretion [34, 35]. Based on this, the use of Metformin in ADPKD patients could decrease energy intake, stimulate AMPK activation and produce mTOR pathway inhibition, which promotes inhibition of cystogenesis and weight gain, the latter having in turn a favorable effect on disease progression and kidney function. In our patients, the slower decline in eGFR compared to other studies could be explained by the concomitant weight loss.

Our study has several limitations. The absence of a control group cannot distinguish between the effect of the treatment and the natural evolution of the disease. The reason that we chose a single-arm design was that we mainly wanted to point out preliminary results regarding the safety and tolerability of Metformin in ADPKD patients. Other limitations are the small sample size and the single-center study enrollment. Another important limitation of the study is the high rate of drop-out (54%), which requires cautious interpretation of tolerability and safety results. Regarding the 12 patients that were lost to follow-up, we do not have any information whether their decision to drop-out was based on medication intolerance or adverse reactions or not, which may lead to an under-estimation of tolerability. The strengths of our study were the prospective design, the intention-to-treat analysis, the length of the follow-up period and the inclusion of patients with advanced CKD stages. Given the small sample size, absence of a control arm and high rate of drop-out we consider that our findings need to be evaluated in a large randomized controlled trial.

## Conclusion

In conclusion, the results from this single-arm pilot study showed that Metformin was well tolerated, with a good safety profile in patients with ADPKD, even in those with advanced CKD and it was not associated with change in eGFR or BMI across the 24 months of follow-up.

## Data Availability

The datasets used and/or analysed during the current study are available from the corresponding author on reasonable request.

## Abbreviations

ADPKD: Autosomal dominant polycystic kidney disease
AMPK: Adenosine monophosphate-activated protein kinase
BMI: Body mass index
cAMP: Cyclic adenosine monophosphate
CFTR: Chloride channel cystic fibrosis transmembrane conductance regulator
CKD: Chronic kidney disease
eGFR: Estimated glomerular filtration rate
ERA-EDTA: European Renal Association-European Dialysis and Transplant Association
ITT: Intention to treat
JAK/STAT: Just another kinase/ signal transducer and activator of transcription
MAPK/ERK: Mitogen-activated protein kinase/extracellular signal-regulated kinase
PP: Per protocol
RRT: Renal replacement therapy
TSC2: Protein of tuberous sclerosis 2

## References

[1] Torres VE, Harris PC, Pirson Y (2007). Autosomal dominant polycystic kidney disease. Lancet..

[2] Solazzo A, Testa F, Giovanella S (2018). The prevalence of autosomal dominant polycystic kidney disease (ADPKD): a meta-analysis of European literature and prevalence evaluation in the Italian province of Modena suggest that ADPKD is a rare and underdiagnosed condition. PLoS One.

[3] Willey CJ, Blais JD, Hall AK (2017). Prevalence of autosomal dominant polycystic kidney disease in the European Union. Nephrol Dial Transplant.

[4] Iliuta IA, Kalatharan V, Wang K (2017). Polycystic kidney disease without an apparent family history. J Am Soc Nephrol.

[5] Cornec-Le Gall E (2018). Genkyst study group; HALT progression of polycystic kidney disease group; consortium for radiologic imaging studies of polycystic kidney disease, Harris PC. Monoallelic mutations to DNAJB11 cause atypical autosomal-dominant polycystic kidney disease. Am J Hum Genet.

[6] Ong AC, Harris PC (2005). Molecular pathogenesis of ADPKD: the polycystin complex gets complex. Kidney Int.

[7] Malekshahabi T, Khoshdel Rad N, Serra AL, et al. Autosomal dominant polycystic kidney disease: disrupted pathways and potential therapeutic interventions. J Cell Physiol. 2019;15. 10.1002/jcp.28094 PMC13484091 30644092

[8] Lanktree MB, Chapman AB (2017). New treatment paradigms for ADPKD: moving towards precision medicine. Nat Rev Nephrol.

[9] Takiar V, Nishio S, Seo-Mayer P (2011). Activating AMP-activated protein kinase (AMPK)slows renal cystogenesis. Proc Natl Acad Sci U S A.

[10] Chang MY, Ma TL, Hung CC, et al. Metformin Inhibits Cyst Formation in a Zebrafish Model of Polycystin-2 Deficiency. Sci Rep. 2017;7(1):7161. Published 2017 Aug 2. 10.1038/s41598-017-07300-x PMC5541071 28769124

[11] Schrier RW, Brosnahan G, Cadnapaphornchai MA (2014). Predictors of autosomal dominant polycystic kidney disease progression. J Am Soc Nephrol.

[12] Nowak KL, You Z, Gitomer B (2018). Overweight and obesity are predictors of progression in early autosomal dominant polycystic kidney disease. J Am Soc Nephrol.

[13] Hui F, Zhang Y, Ren T, et al. Role of metformin in overweight and obese people without diabetes: a systematic review and network meta-analysis. Eur J Clin Pharmacol. 2018;3. 10.1007/s00228-018-2593-3 30511328

[14] Pei Y (2009). Unified criteria for ultrasonographic diagnosis of ADPKD. J Am Soc Nephrol.

[15] Revicki DA, Wood M, Wiklund I (1998). Reliability and validity of the gastrointestinal symptom rating scale in patients with gastroesophageal reflux disease. Qual Life Res.

[16] Garber AJ, Duncan TG, Goodman AM (1997). Efficacy of metformin in type II diabetes: results of a double-blind, placebo-controlled, dose-response trial. Am J Med.

[17] Lord JM, Flight IH, Norman RJ (2003). Metformin in polycystic ovary syndrome: systematic review and meta-analysis. BMJ..

[18] Sallée M, Rafat C, Zahar JR (2009). Cyst infections in patients with autosomal dominant polycystic kidney disease. Clin J Am Soc Nephrol.

[19] Bolen Shari, Feldman Leonard, Vassy Jason, Wilson Lisa, Yeh Hsin-Chieh, Marinopoulos Spyridon, Wiley Crystal, Selvin Elizabeth, Wilson Renee, Bass Eric B., Brancati Frederick L. (2007). Systematic Review: Comparative Effectiveness and Safety of Oral Medications for Type 2 Diabetes Mellitus. Annals of Internal Medicine.

[20] Moen MF, Zhan M, Hsu VD (2009). Frequency of hypoglycemia and its significance in chronic kidney disease. Clin J Am Soc Nephrol.

[21] The EuropeanMedicines Agency (EMA). Use of metformin to treat diabetes now expanded to patients with moderately reduced kidney function: recommendations for patients with kidney impairment updated in product information [Internet], 2016.Available from http://www.ema.europa.eu/docs/en_GB/document_library/Press_release/2016/10/WC500214248.pdf. Accessed 5 Jan 2019.

[22] The U.S. Food and Drug Administration (FDA). Drug Safety Communication: FDA revises warnings regarding use of the diabetes medicine metformin in certain patients with reduced kidney function [Internet], 2016. Available from https:// www.fda.gov/downloads/Drugs/DrugSafety/ UCM494140.pdf. Accessed 5 Jan 2019.

[23] Salpeter SR, Greyber E, Pasternak GA (2010). Risk of fatal and nonfatal lactic acidosis with metformin use in type 2 diabetes mellitus (review). Cochrane Database Syst Rev.

[24] Ekström, Nils et al. Effectiveness and safety of metformin in 51 675 patients with type 2 diabetes and different levels of renal function: a cohort study from the Swedish National Diabetes Register. *BMJ open* vol. 2,4 e001076. 13 Jul. 2012. 10.1136/bmjopen-2012-001076 PMC3400073 22798258

[25] Duong JK, Roberts DM, Furlong TJ (2012). Metformin therapy in patients with chronic kidney disease. Diabetes Obes Metab.

[26] Lalau JD, Kajbaf F, Bennis Y (2018). Metformin treatment in patients with type 2 diabetes and chronic kidney disease stages 3A, 3B, or 4. Diabetes Care.

[27] Tirosh A (2013). Renal function following three distinct weight loss dietary strategies during 2 years of a randomized controlled trial. Diabetes Care.

[28] Pisani A, Riccio E, Bruzzese D (2018). Metformin in autosomal dominant polycystic kidney disease: experimental hypothesis or clinical fact?. BMC Nephrol.

[29] Torres VE, Chapman AB, Devuyst O (2012). TEMPO 3:4 trial Investigators.Tolvaptan in patients with autosomal dominant polycystic kidney disease. N Engl J Med.

[30] Torres VE, Chapman AB, Devuyst O (2017). REPRISE trial Investigators.Tolvaptan in later-stage autosomal dominant polycystic kidney disease. N Engl J Med.

[31] Serra AL, Poster D, Kistler AD (2010). Sirolimus and kidney growth in autosomal dominant polycystic kidney disease. N Engl J Med.

[32] Braun WE, Schold JD, Stephany BR (2014). Low-dose rapamycin(sirolimus) effects in autosomal dominant polycystic kidney disease: an open-label randomized controlled pilot study. Clin J Am Soc Nephrol.

[33] Myint TM, Rangan GK, Webster AC (2014). Treatments to slow progression of autosomaldominant polycystic kidney disease: systematic review and meta-analysis ofrandomized trials. Nephrology (Carlton).

[34] Jia G, Aroor AR, Martinez-Lemus LA (2014). Overnutrition, mTOR signaling, and cardiovascular diseases. Am J Physiol Regul Integr Comp Physiol.

[35] Dann Stephen G., Selvaraj Anand, Thomas George (2007). mTOR Complex1–S6K1 signaling: at the crossroads of obesity, diabetes and cancer. Trends in Molecular Medicine.

